# Risk Prediction of Ureaplasma urealyticum Affecting Sperm Quality Based on Mathematical Model and Cross-Sectional Study

**DOI:** 10.1155/2022/2498306

**Published:** 2022-05-25

**Authors:** Huang Liu, Kai Yang, Liping He, Shenghui Zhu, Tao Pang, Zhiyong Zhu, Yunyi Yao, Houbin Zheng, Qingqi Zeng, Xinzong Zhang

**Affiliations:** ^1^The First School of Clinical Medicine, Nanjing University of Chinese Medicine, Nanjing 210023, China; ^2^Department of Andrology, NHC Key Laboratory of Male Reproduction and Genetics, Guangdong Provincial Reproductive Science Institute (Guangdong Provincial Fertility Hospital), Human Sperm Bank of Guangdong Province, Guangzhou 510060, China; ^3^Department of Andrology, Zhangjiagang TCM Hospital Affiliated to Nanjing University of Chinese Medicine, Zhangjiagang 215600, China; ^4^Clinical Laboratory Center, NHC Key Laboratory of Male Reproduction and Genetics, Guangdong Provincial Reproductive Science Institute (Guangdong Provincial Fertility Hospital), Human Sperm Bank of Guangdong Province, Guangzhou 510060, China; ^5^Department of Integrated Chinese and Western Medicine, Jiangsu Health Vocational College, Nanjing 211800, China

## Abstract

**Objective:**

To explore the risk of Ureaplasma urealyticum (*UU*) affecting sperm quality.

**Methods:**

Prospective cross-sectional study was conducted. In total, 340 semen samples were collected. According to whether they were infected with UU, the samples were divided into the UU-positive group (*observation group*) and UU-negative group (*control group*). The patients with UU-positive were followed up to obtain treatment and collected the semen again after treatment. The semen characteristics and sperm parameters were detected and compared, and the relationship of UU and the sperm quality was analyzed by mathematical models.

**Results:**

There were 104 UU-positive semen samples in all, with an overall infection rate of 30.6%, which was highest in 31 to 40-year-old men, and over 40-year-old men were the lowest. The pH, PR, VCL, VSL, and STR in the observation group were significantly lower than those in the control group (*allP* < 0.001), while SV, NP, and WOB were significantly higher (*allP* < 0.001). After treatment, the pH, VSL, LIN, WOB, and STR in the observation group were significantly higher than before (*allP* < 0.001), while SV and VCL were significantly lower (*allP* < 0.001). UU infection was closely correlated with pH, PR, NP, VCL, VSL, WOB, and STR. During the treatment, pH, PR, VSL, WOB, and STR increased, but NP and VCL decreased. 7 major factors that would affect SQ were extracted, of which VAP, LIN, and UU were the first three main factors. The risk of SQ declining after UU infection increased nearly twice with the change of PR and VCL and increased 0.08 times with STR.

**Conclusion:**

UU may approximately double the risk of altering the sperm's curvilinear movement rate and straightness to affect the sperm quality.

## 1. Introduction

Studies have shown that there is an increasing number of reproductive tract infections in men of reproductive age globally [1]. Male fertility is declining year by year, and it is endangered by inflammation [2]. Mycoplasma (contained: Ureaplasma urealyticum (*UU*), Mycoplasma hominis (*MH*), Mycoplasma genitalium (*MG*)), chlamydia, and gonorrhea are the most common pathogens causing genital tract infections [3]. In particular, mycoplasma infection has a high infection rate and usually does not cause serious clinical symptoms [4]. It is very hidden and often occurs together with other diseases, which is especially easy to ignore [5]. Therefore, how to prevent and treat mycoplasma infection early is a hot spot and focus of male reproductive health research.

Ureaplasma urealyticum (*UU*) is a symbiotic microorganism of the human reproductive tract that has the highest infection rate among mycoplasma [6]. It can be carried out by normal people without causing disease. When the internal environment of the body changes and resistance decreases, it will overrepresent and cause disease [7]. Studies have shown that UU can cause prostatitis or epididymitis in men [8], which can produce toxic substances that interfere with fertilization [9], and it may cause female reproductive tract infection and fetal damage [10], as well as hinder sperm movement [11]. However, the mechanism by which UU affects sperm parameters is not clear at present. Some studies believe that UU interferes with sperm tail motility through adhesion [12], while others believe that UU affects sperm motility through an immune response [13]. In order to explore the risk of Ureaplasma urealyticum (*UU*) affecting sperm quality (*SQ*), we conducted a prospective cross-sectional study and mathematical model analysis and found surprising results, which might provide a clinical reference.

## 2. Materials and Methods

### 2.1. Sample Sources

All samples were donated by patients who came to the outpatient Department of Andrology of Guangdong Provincial Reproductive Science Institute (*Guangdong Provincial Fertility Hospital*) from January 2020 to December 2020. The random sampling method was utilized, and the sample size was estimated based on the following equation with *α* = 0.05, *P* = 25% [6, 14], *Z*_1−*α*/2_ = 1.96, *ɗ* = 0.05. We selected 340 fertile men with a 20% risk of loss and divided into two groups with 170 patients in each. According to the results of UU culture and drug sensitivity tests, the samples were divided into a UU-positive group (*Observation group*) and a UU-negative group (*Control group*),

(1) $$\begin{array}{c} N=\frac{{Z^{2}}_{1-\alpha /2}p\left(1-p\right)}{{ɗ}^{2}}. \end{array}$$

### 2.2. Patients' Data

The patients were 21~46 years old, with an average age of 33.1 ± 6.9 years old. Among the men, 218 had primary infertility, 71 had secondary infertility, and 51 had a physical examination before childbirth. They were scheduled for semen screening, with microbial cultures of semen (*including UU*, *Chlamydia*, *and Neisseria gonorrheae*) as routine tests for fertility assessment. Informed consent was obtained from the patients. At the same time, the approval and consent of the ethics committee of Guangdong Provincial Reproductive Science Institute (*Guangdong Provincial Fertility Hospital*) was also obtained (No. [2020] (28)).

### 2.3. Inclusion Criteria

All semen samples were obtained by external ejaculation after masturbation in the semen collection room in the hospital at 25°C. Patient information and data were complete, and follow-up requirements.

### 2.4. Exclusion Criteria

Those who did not meet the inclusion criteria had used antibiotics in the two weeks before the test, had systemic diseases, had a long-term drug use history, had a history of exposure to radioactive substances or toxic substances, had chromosomal abnormalities, had azoospermia factor (*AZF*) gene deletion, had varicocele, had azoospermia, or had testicular tumors. Their spouses had abnormal physical examinations.

### 2.5. Semen Analysis

Semen ejaculated from the patient after 2-7 days of abstinence was collected and placed in a water bath at 37°C for liquefaction. The computer-aided semen analysis (*CASA*) system was used to measure sperm parameters according to the World Health Organization (*WHO*) guidelines for semen analysis (*5^th^ edition, 2010*) [15]. The data were analyzed with an SCA automatic sperm quality analyzer (*SCA-05, Spain, registration certificate no. 20172706051*).

### 2.6. UU Detection

All samples were detected by one-body kit for UU cultivation of drug sensitivity: (contained culture medium for identification of mycoplasma (*culture method*, *fixed medium for Mycoplasma*, *20 people*, *product number 20172400944*). Mycoplasma reagent combination (*culture method, product number 20172400952*)) (*Jiangmen Caring Trading Co., Ltd., Guangdong, China*). The steps are as follows: ① add 55 *μ*l culture medium into the first well on the upper left of the kit as negative control; ② take 200 *μ*l semen sample, add it to culture medium, and stir thoroughly; ③ absorb 55 *μ*l culture liquid mixed with specimens and drop it into mycoplasma identification medium (*A7 petri dish*); ④ add each 55 *μ*l of culture medium mixed with specimens into the remaining holes of the drug-sensitive plate; and ⑤ cover each hole with paraffin oil and incubate at 37°C for 48 hours to observe the results. A research grade universal microscope (*BX51T-12P01, Olympus, Japan*) was used to observe the colonies of UU.

### 2.7. Follow-Up and Treatment

The patients with UU positive (*observation group*) were followed up and treated with sensitive antibiotics according to the drug sensitivity test results for 2 weeks. Doxycycline hydrochloride (*Xianqiang Pharmaceutical Co., Ltd., Guangdong,*0.1 g × 12 s) was used for the treatment of UU, with 0.1 g each time and twice a day, which lasted 14 days as one course of the treatment. After that, the patients were followed up to collect the semen sample again by stopping the drug for 1 week. All of them were advised to avoid sexual life, and they kept ejaculation 1 ~ 2 times during the treatment.

### 2.8. Observation Index

The indexes of semen volume (*SV*), pH value (*pH*), sperm concentration (*SC*), sperm progressive motility (*PR*), non-progressive motility (*NP*), immotility (*IM*), normal forms (*N*), curvilinear velocity (*VCL*), straight-line (rectilinear) velocity (*VSL*), average path velocity (*VAP*), and the linearity (*VSL/VCL, LIN*), wobble (*VAP/VCL, WOB*), and straightness (*VSL/VAP, STR*) were observed as the sperm quality (*SQ*) according to the World Health Organization (*WHO*) guidelines for semen analysis (*5^th^ edition, 2010*) [14] (the normal reference range was referred to Supplementary Table 1).

### 2.9. Statistical Analysis

Statistical analysis was conducted using *SPSS 19.0 (IBM Corp. Version 19.0. Armonk, NY, USA)*. Measurement data is expressed as the $\text{mean }\left(\bar{x}\right)\pm \text{standard deviation }\left(\text{s}.\text{d}.\right)$, and the counting data are expressed as percentages (%) and rates. The *Kolmogorov–Smirnov test* (*K-S* test) was used to test the normality of the data. If the data were assumed to be *normally distributed*, *one-way ANOVA* was used to test the homogeneity of variance, and *Student'st*-test and the *chi-square test* (*χ*^2^ test) were used to test the obtained parameters. If the data were assumed to be abnormally distributed, the *Mann–WhitneyUrank-sum test* (*Wilcox* test) was used. *Spearman correlation analysis* was used to analyze the correlation between the data. *Linear regression analysis* was used to judge the trend of change. *Factor analysis* was used to evaluate the main components of the impact. *Cox regression analysis* was used to predict the proportion of risk. *P* < 0.05 was considered statistically significant.

## 3. Results

### 3.1. Baseline Characteristics

104 UU-positive semen samples (*observation group*) and 236 UU-negative samples (*control group*) had been collected in our study. The patient information and sample data of the two groups were tested by *Kolmogorov–Smirnov (K-S)* normal distribution for normality, and it was found that the BMI, SV, SC, IM, VCL, VSL, and VAP were in line with normal distribution, while the age, height, weight, abstinence time (*AT*), pH, PR, NP, N, LIN, WOB, and STR were not (Table 1). *One-way ANOVA* found that the variance in BMI, SC, VSL, and VAP were uniform, but the variance in SV, IM, and VCL was uneven (Table 2). Although the abstinence time of the observation group was slightly lower than that of the control group, there were no significant differences in age, height, weight, BMI, AT, marriage status, and fertility status between the two groups (Table 3).

### 3.2. UU Infection Rate

**Figure 1:**
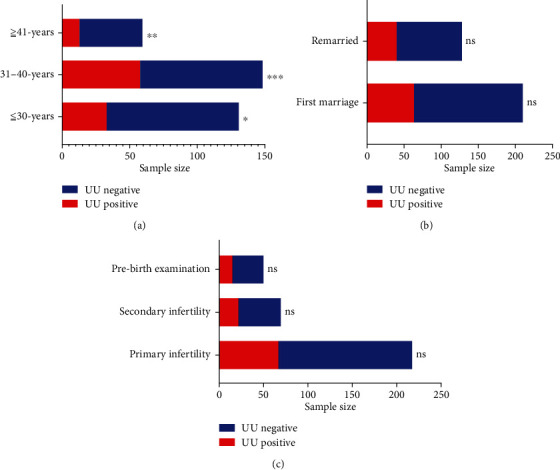
(a) Comparison of UU infection rates in different age groups: 30-40 years vs. <30 years: ^∗∗∗^*P* < 0.001. 30-40 years vs. >41 years: ^∗∗^*P* < 0.01. >40-yeas vs. <30 years: ^∗^*P* < 0.05. (b) Comparison of UU infection rate in different marriage status: remarriage vs. first marriage. (c) Comparison of UU infection rates in different fertility states: primary infertility vs. secondary infertility vs. prefertility physical examination.

There were 104 UU-positive semen samples in all 340 samples, with an overall infection rate of 30.6%. The UU infection rate was significantly different in the different age groups, UU infection rates (*38.93%*) were highest in the 31 to 40 years age group, the second was the 30 years or less group with the UU infection rate of 25.19%, the over 40 age group had the lowest (*21.67%*) (*P* < 0.001) (Figure 1(a)), while there was no difference in the UU infection rate between the different marriage status (*P* > 0.005) (Figure 1(b)) and different fertility status (*P* > 0.005) (Figure 1(c)).

### 3.3. Semen Parameters

**Figure 2:**
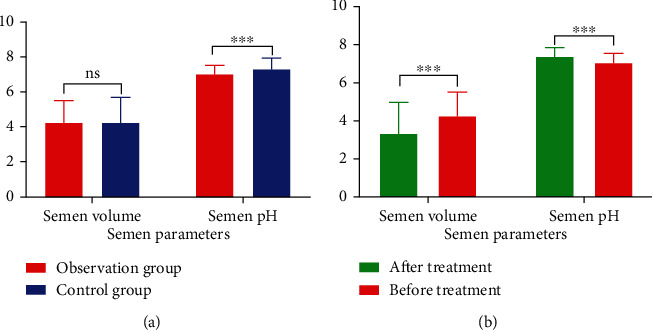
Comparison of semen parameters: (a) comparison between the treatment group and the observation group: SV: no difference, pH: *P* < 0.01. (b) Comparison of the observation group before and after treatment: SV and pH: all *P* < 0.01.

The SV of the observation group was slightly higher than that of the control group, but there was no difference (*P* > 0.005), while the pH was significantly lower than that of the control group, and the difference was statistically significant (*P* < 0.001) (Figure 2(a)). The SV after treatment in the observation group was significantly lower than that before treatment (*p <0.001*), and the pH was significantly higher than before treatment (*P* < 0.001), with statistically significant differences (*P* < 0.001) (Figure 2(b)).

### 3.4. Sperm Parameters

**Figure 3:**
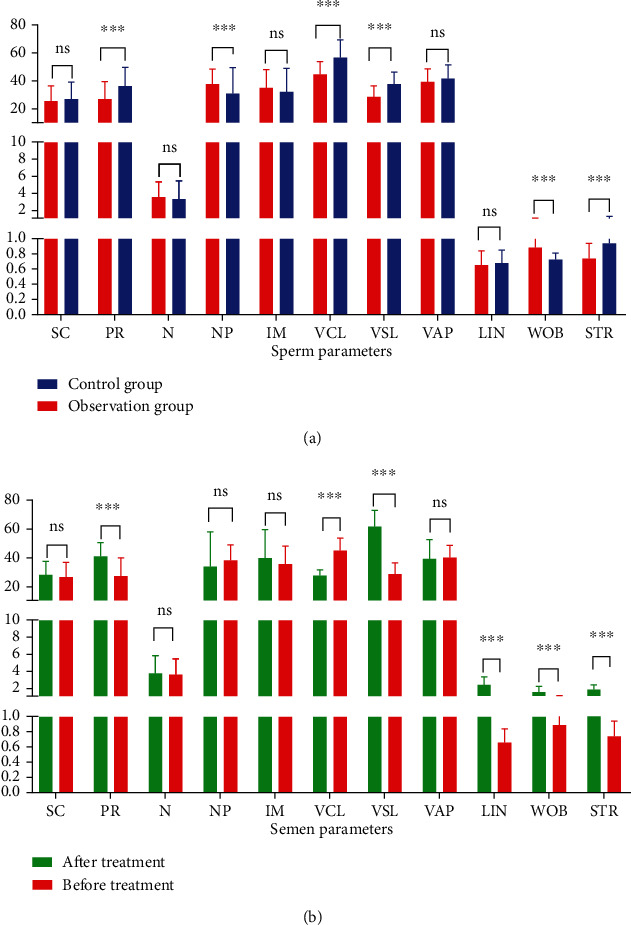
Comparison of sperm parameters: (a) comparison between the observation group and the control group: PR, NP, VCL, VSL, WOB, and STR: all *P* < 0.01. (b) Comparison of observation group before and after treatment: PR, VCL, VSL, LIN, WOB, and STR: all *P* < 0.01.

The sperm PR, VCL, VSL, and STR in the observation group were significantly lower than those in the control group (*P* < 0.001), while NP and WOB were significantly higher (*P* < 0.001), but there was no difference in SC, N, IM, VAP, and LIN (*P* > 0.005) (Figure 3(a)). After treatment, sperm PR, VSL, LIN, WOB, and STR in the observation group were significantly higher than before (*P* < 0.001), while VCL was significantly lower (*P* < 0.001), and the SC, N, NP, IM, and VAP showed no difference (*P* > 0.005) (Figure 3(b)).

### 3.5. Correlation between UU and SQ

**Figure 4:**
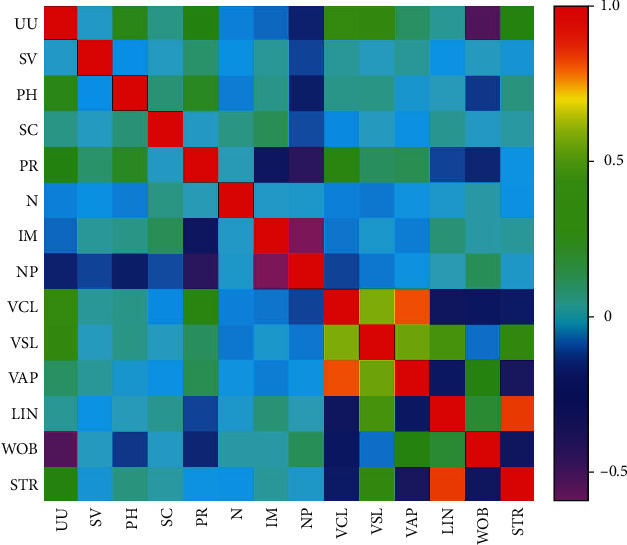
Correlation between UU and SV, pH, SC, PR, NP, IM, N, VCL, VSL, VAP, LIN, WOB, and STR.

Spearman correlation analysis showed that UU infection was closely correlated with pH (*r* = 0.207, *P* < 0.001), PR (*r* = 0.325, *P* < 0.001), NP (*r* = −0.191, *P* < 0.001), VCL (*r* = 0.449, *P* < 0.001), VSL (*r* = 0.446, *P* < 0.001), WOB (*r* = 0.574, *P* < 0.001), and STR (*r* = 0.305, *P* < 0.001), but there was no relation with SV (*r* = −0.005, *P* = 0.928), SC (*r* = 0.063, *P* = 0.244), N (*r* = −0.063, *P* = 0.247), IM (*r* = −0.084, *P* = 0.124), VAP (*r* = 0.099, *P* = 0.067), and LIN (*r* = 0.054, *P* = 0.325) (Figure 4).

### 3.6. Change Trend before and after Treatment

**Figure 5:**
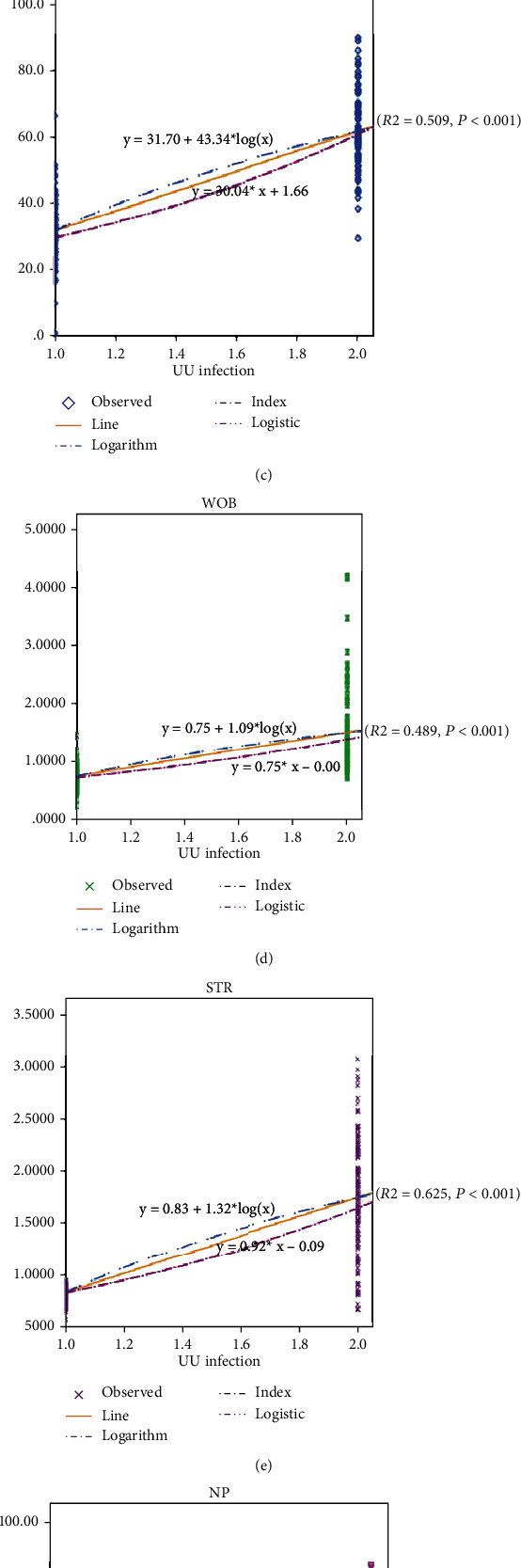
UU infection and the trend of various indicators: (a) UU and pH, (b) UU and PR, (c) UU and VSL, (d) UU and WOB, (e) UU and STR, (f) UU and NP, and (g) UU and VCL.

Time-series trend analysis was used to evaluate changes in related indicators before and after treatment of UU infection. The results indicated that pH (Figure 5(a)), PR (Figure 5(b)), VSL (Figure 5(c)), WOB (Figure 5(d)), and STR (Figure 5(e)) increased with the progress of treatment, while NP (Figure 5(f)) and VCL (Figure 5(g)) decreased with the progress of treatment.

### 3.7. Factor Analysis

**Figure 6:**
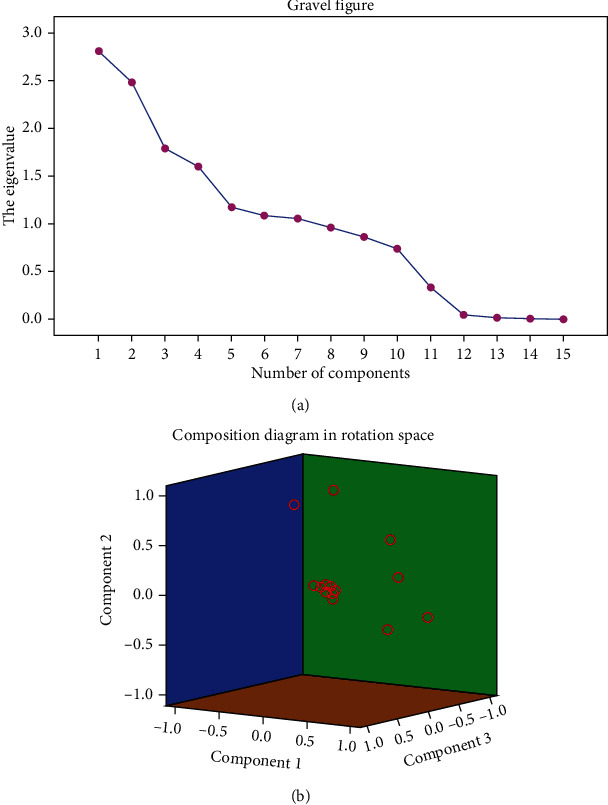
Principal factor analysis: (a) major factor extraction lithotriptic diagram. (b) Major factor component diagram.

Principal factor analysis was used to evaluate the related components affecting SQ after UU infection. The results indicated that 7 major factors affecting SQ could be extracted from the 15 factors of UU, AT, SV, pH, SC, PR, NP, IM, N, VCL, VSL, VAP, LIN, WOB, and STR (Figure 6(a)). Kaiser standardization was adopted by the orthogonal rotation method; it was found that VAP, LIN, and UU are the first three main factors (Figure 6(b)).

### 3.8. Cox Regression Analysis

**Figure 7:**
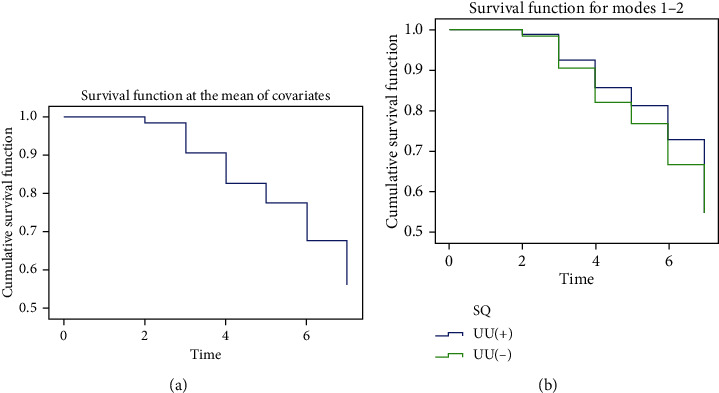
Cox regression analysis: (a) covariate survival function and (b) SQ classification survival function. SQ: sperm quality: (1) normal sperm quality and (2) abnormal sperm quality.

AT was taken as the time variable, and UU infection was taken as the state variable, and SQ was taken as the classification variable. The *COX regression analysis* showed that PR, VCL, and STR were the independent factors affecting SQ after UU infection (*P* < 0.001) (Tables 4(a) and 4(b). Compared with the normal population, the risk of SQ decline after UU infection increased nearly twice with the change of PR and VCL with the progress of abstinence (HR = 0.980 (95% CI: 0.964-0.996); HR = 0.940 (95% CI: 0.922-0.957)) and increased 0.08 times with STR (HR = 0.082 (95% CI: 0.033-0.204)) (Figures 7(a), 7(b)).

## 4. Discussion

Human semen is a mixed suspension containing the secretion of male reproductive tract accessory gonadal organs. Its main components are sperm and seminal plasma. Seminal plasma accounted for more than 90% [16]. Some specific cytokines, protein components, or glycopeptides in seminal plasma could predict and analyze the physiological and pathological functions of the specific accessory gonad [17]. The content or concentration changes of these biological components would also directly affect the biological characteristics of semen, thus directly or indirectly affecting sperm parameters and male fertility [18, 19]. At present, studies have shown that semen liquefaction is regulated by coagulation and liquefaction factors [20]. Coagulation factors, such as semen coagulating protein, collagen, or fibronectin, could keep the gem from ejaculating semen and make the semen thick [21]. Liquefied factors composed of prostate-specific antigens, fibrinolytic enzymes, and acid phosphatase could promote the development of semen liquefaction [22]. A study also believed that after genital tract infection, the occurrence of inflammation leads to abnormal pH values of semen and changes in the physical and chemical properties of semen [23]. Therefore, studying the changes in semen traits was helpful for judging the quality of sperm. We selected UU-positive-infected samples and negative uninfected samples. We found that there were some abnormal changes in semen parameters and sperm quality in semen samples after infection. This suggests that the increased secretion of epithelial cells of genital accessory gonadal organs caused by inflammation and infection, especially the secretion of inflammatory substances, will change the proportion of semen and seminal plasma components, leading to changes in the physical and chemical properties of semen and then affecting the quality of sperm.

**Figure 8:**
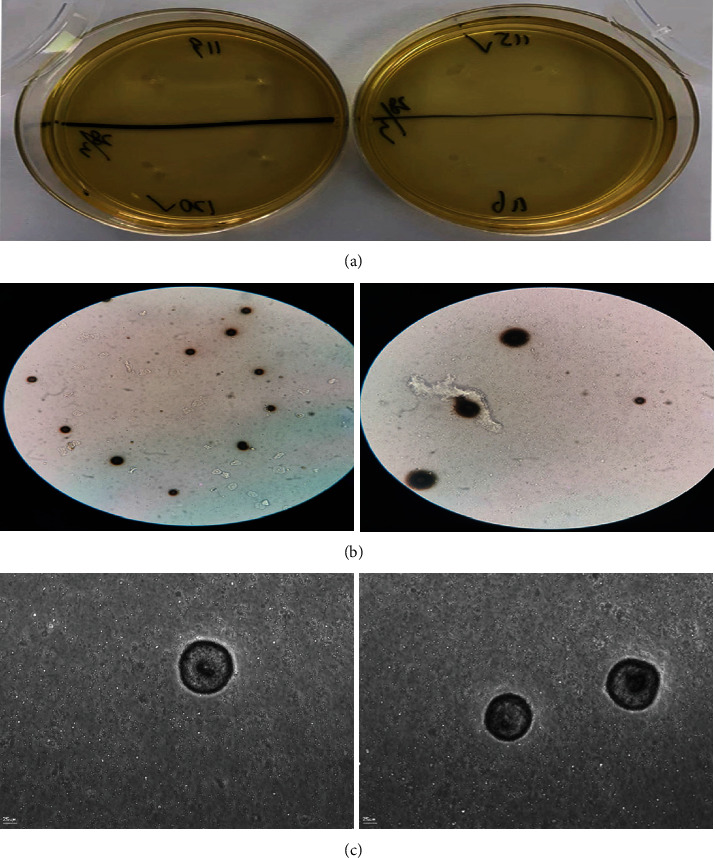
UU image. (a) UU grown on culture medium. (b) UU under ordinary light microscope. (c) UU under ultrahigh definition microscope.

We analyzed the change in semen volume and pH and found that the semen volume increased significantly after UU infection, but pH declined. We think this might be the result of gonad function secretion increasing. Especially when the inflammatory stimulation produced by UU infection acted on the prostate, the function of the prostate would be impaired, and the secretion of citric acid would decrease; at this time, the acid-base balance of semen would be unbalanced [24], so the pH of semen and sperm motility would decrease in the acidic environment. Therefore, the samples we observed after the UU infection must have been mixed with more prostate fluid, so the semen volume increased. Another possibility was that the pH of prostatic fluid was low, so the excessive secretion of prostatic fluid had an impact on the semen pH, resulting in the decrease of semen pH, and also leading to an unsuitable microenvironment for the sperm. So, sperm vitality would be weakened, and thus, quality would decrease. This phenomenon was also found in the process of UU culture of semen. Disordered sperm aggregation appeared near the UU cluster in the medium, while the sperm was arranged more orderly at a distance from the UU (Figures 8(a)–8(c)).

Research shows that UU is the most common type of male genital tract infection at present, with no obvious symptoms, high concealment, and great potential harm, so it is listed as one of the most easily neglected fertility killers [25]. UU might activate the immune response *in vivo* and enhance the chemotaxis and stress effects of inflammatory cells such as neutrophils [26]. After UU infection, the epithelial cell membrane of the genital tract mucosa in males adhered and was destroyed. At the same time, a large number of ammonia substances would be produced, which would produce toxic effects on the genital tract epithelium. In serious cases, it easily causes the adhesion and migration of inflammatory substances and leads to genital tract obstruction [27]. A study found that a decrease in sperm parameters, sperm necrosis, DNA denaturation, an increase in intracellular ROS, and a decrease in MMP were related to an increase in leukocyte elastase [28]. A similar situation was also found in our observation. We found that the sperm motility in UU-positive semen samples was significantly lower than that in UU-negative semen samples.

In particular, the forward and oscillating characteristics of sperm were studied. We studied the curvilinear and linear rates of sperm (*VCL and STR*). We found that although there was no significant difference in VAP of sperm after UU infection, VSL decreased significantly, which indicated that the sperm's forward orientation was affected. Previous studies have shown that elastase can stick to the tail of sperm, affecting sperm motility [29]. When the sperm infected with UU was like the fish was caught by the tail, it could not be straightforward but could only be away from side to side. So, the WOB of sperm infected with UU will increase. When we applied sensitive antibiotics to inactivate UU, the therapeutic effect corroborates our analysis, the WOB of the sperm significantly decreased and straightness significantly increased.

Mathematical modeling is of great help to medicine [30, 31]. Through effective mathematical model analysis, the risk of disease occurrence or intervention can be predicted [32, 33]. For further in-depth analysis of the cause of the decline in sperm quality, we used a trend analysis model, principal factor analysis model, and Cox regression risk ratio model to evaluate the risk of sperm quality affected by UU infection. Our valid data suggested that UU infection might cause significant changes in sperm motility parameters, especially in sperm motility forward performance, between UU-positive samples and UU-negative samples, and before and after UU treatment. We found that UU infection increased the risk of sperm motility impairment by about twice that of the normal population. We hope that this might provide a useful reference for clinical prevention, early diagnosis, and treatment of UU infection.

In conclusion, our study revealed that although the clinical symptoms of UU were concealed and difficult to detect, UU could promote the secretion of accessory gonadal to affect sperm motility, which may be reflected in the changes in semen traits early and may be used to predict the presence of UU infection.

Due to the simplicity of the detection content methods, our finding may be a new potential target or possible mechanism of UU affecting sperm motility for the first time preliminarily, which would bring important inspiration to our later research. However, we will continue more in-depth studies to further explore its mechanism from the perspective of molecular biology.

## Figures and Tables

**Table 1:** Kolmogorov-Smirnov test.

Indicator	*Z*	*P*
Age (y)	1.439	0.032
Height (cm)	2.777	<0.001
Weight (kg)	3.337	<0.001
BMI (kg · m^−2^)	0.817	0.518
AT (d)	3.202	<0.001
SV (ml)	0.893	0.402
pH	1.713	0.006
SC (×10^6^ ml^−1^)	1.320	0.061
PR (%)	1.406	0.038
N (%)	1.711	0.006
NP (%)	2.423	<0.001
IM (%)	1.023	0.246
VCL (*μ*m S^−1^)	0.708	0.698
VSL (*μ*m S^−1^)	0.736	0.651
VAP (*μ*m S^−1^)	0.862	0.447
LIN	1.965	0.001
WOB	1.616	0.011
STR	5.211	<0.001

Note: BMI: body mass index; AT: abstinence time; SV: semen volume; pH: pH value; SC: sperm concentration; PR: sperm progressive motility; N: normal forms; NP: nonprogressive motility; IM: immotility; VCL: curvilinear velocity; VSL: straight-line (rectilinear) velocity; VAP: average path velocity; LIN: linearity (VSL/VCL); WOB: wobble (VAP/VCL); and STR: straightness (VSL/VAP) (World Health Organization. 2010, 5^th^, ed).

**Table 2:** Homogeneity of variance test.

Indicator	Levene statistic	*df*1	*df*2	*P*
BMI	1.037	1	338	0.309
SV	3.903	1	338	0.049
SC	0.116	1	338	0.734
IM	7.420	1	338	0.007
VCL	10.664	1	338	0.001
VSL	1.985	1	338	0.160
VAP	1.740	1	338	0.188

Note: BMI: body mass index, SV: semen volume; SC: sperm concentration; IM: immotility; VCL: curvilinear velocity; VSL: straight-line (rectilinear) velocity; VAP: average path velocity.

**Table 3:** Baseline characteristics.

Indicator	Observation group	Control group	*P*
Sample size (*n*)	104	236	—
Age (y), median (*Q*_25_ ~ *Q*_75_)	33.34 (29.00~38.00)	32.99 (27.00~39.00)	0.669*^Δ^*
Height (cm), median (*Q_25_ ~ Q_75_*)	166.31 (163.01~169.00)	165.86 (162.00~169.00)	0.367*^Δ^*
Weight (kg), median (*Q*_25_ ~ *Q*_75_)	66.92 (60.50~70.80)	66.63 (60.40~70.80)	0.689*^Δ^*
BMI (kg m^−2^), mean ± s.d.	24.22 ± 2.39	24.26 ± 2.57	0.880∗
AT (d), median (*Q*_25_ ~ *Q*_75_)	4.01 (3.00~5.00)	4.17 (3.00~5.00)	0.358*^Δ^*
Marriage status, *n* (%)			
First marriage	64 (61.5)	147 (62.3)	0.494^☆^
Remarried	40 (38.5)	89 (37.7)	
Fertility status, *n* (%)			
Primary infertility	67 (64.4)	151 (64.0)	0.980^☆^
Secondary infertility	22 (21.2)	49 (20.8)	
Prebirth physical examination	15 (14.4)	36 (15.2)	

Note: BMI: body mass index; AT: abstinence time. *P* values were derived from Student's *t*-test for parametric comparisons and the Mann–Whitney *U* test and Chi-square test for nonparametric comparisons. *Q*_25_: 25^th^ percentile; *Q*_75_: 75^th^ percentile; s.d.: standard deviation. ^∗^Student's *t*-test. *^Δ^*Mann–Whitney *U* test. ^☆^*Chi-square test*.

**Table:** (a) Comprehensive test of model coefficients^a^

Step	-2 log likelihood	Overall (score)			Change from previous block		
		Chi-square	*df*	Sig.	Chi-square	*df*	Sig.
1	973.020	105.539	6	0.000	105.539	6	0.000

Note: ^a^beginning block number. 1 method: forward stepwise (likelihood ratio).

**Table:** (b) Variables in an equation

Indicators	*B*	SE	Wald	*df*	Sig.	Exp(*B*)	95.0% CI for Exp(*B*)	
							Lower	Upper
pH	-0.284	0.206	1.903	1	0.168	0.753	0.503	1.127
PR	-0.020	0.008	5.736	1	0.017	0.980	0.964	0.996
NP	0.003	0.008	0.138	1	0.710	1.003	0.987	1.019
VCL	-0.062	0.010	42.107	1	0.000	0.940	0.922	0.957
STR	-2.505	0.466	28.851	1	0.000	0.082	0.033	0.204
SQ	-0.249	0.348	0.512	1	0.474	0.780	0.395	1.541

Note: pH: pH value; PR: sperm progressive motility; NP: nonprogressive motility; VCL: curvilinear velocity; STR: straightness (VSL/VAP); SQ: sperm quality (World Health Organization. 2010, 5^th^, ed).

## Data Availability

The relevant sample data used to support the findings of this study are included within the article, which are available from the corresponding author upon request

## Abbreviations

UU: Ureaplasma urealyticum
pH: pH value
PR: Sperm progressive motility
VCL: Curvilinear velocity
VSL: Straight-line (rectilinear) velocity
STR: Straightness
SV: Semen volume
NP: Nonprogressive motility
WOB: Wobble
LIN: The linearity
SQ: Sperm quality
VAP: Average path velocity
MH: Mycoplasma hominis
MG: Mycoplasma genitalium
AZF: Azoospermia factor
CASA: Computer-aided semen analysis
WHO: World Health Organization
SC: Sperm concentration
IM: Immotility
N: Normal forms
K-S: Kolmogorov–Smirnov
AT: Abstinence time.

## Supplementary Materials

Supplementary Materials Supplementary Table 1: reference of the indicators.
