# Supplementary material for: Risk Prediction of Ureaplasma urealyticum Affecting Sperm Quality Based on Mathematical Model and Cross-Sectional Study
Source: Comput Math Methods Med. 2022 May 25;2022:2498306. doi: 10.1155/2022/2498306 (PMC9159871; doi:10.1155/2022/2498306)
Supplement: Supplementary Materials — Supplementary Table 1: reference of the indicators. [file 2498306.f1.docx]

Supplementary table 1: Reference of the indicators

| Indicator | Reference range |
| --- | --- |
| SV（ml） | 1.5 (1.4～1.7)^*^ |
| pH | ≥7.2^*^ |
| SC（×10^6^ ml^-1^） | 15 (12～16)^*^ |
| PR（%） | 32 (31～34)^*^ |
| N（%） | 4 (3～4)^*^ |

Note: ^*^ *lower reference limits of semen parameters according to World Health Organization (2010, 5^th^, ed). SV: Semen volume. Ph: pH value. SC: Sperm concentration. PR: Sperm progressive motility. N: Normal forms*
